# Gut microbiota-mediated Th17/Treg regulation in osteoporotic fracture: from postmenopausal dysbiosis to callus repair

**DOI:** 10.3389/fimmu.2026.1918017

**Published:** 2026-09-29

**Authors:** Zixin Xu, Shutao Jin, Yize Liu, Jianjun Li

**Affiliations:** Department of Orthopedic Trauma, Shengjing Hospital of China Medical University, Shenyang, China

**Keywords:** gut microbiota, IL-17 signaling, intestinal barrier, microbial metabolites, osteoimmunology, osteoporotic fracture repair, postmenopausal osteoporosis, Th17/Treg regulation

## Abstract

Osteoporotic fractures are traditionally regarded as severe clinical consequences of low bone mass and impaired bone strength, but fracture risk and repair outcomes are also shaped by systemic inflammation, intestinal barrier integrity, microbial metabolism, and osteoimmune regulation. Increasing evidence indicates that gut dysbiosis associated with postmenopausal osteoporosis is accompanied by increased lipopolysaccharide exposure, altered microbial metabolites, enhanced pro-inflammatory cytokine signaling, and dysregulation of T helper 17 and regulatory T cells. These changes may promote bone resorption before fracture and create an unfavorable immune background for subsequent repair. Fracture studies further show that interleukin-17 signaling, gut-derived T helper 17 cells, γδ T cells, and regulatory T cells participate in callus inflammation, progenitor recruitment, cartilage-to-bone transition, mineralization, and remodeling in a stage- and context-dependent manner. Microbial metabolites, particularly short-chain fatty acids such as butyrate, tryptophan-derived indoles, and bile acid metabolites, can modulate T helper 17/regulatory T-cell homeostasis through immunometabolic and receptor-dependent pathways, whereas trimethylamine N-oxide and polyamines may further modify inflammatory and bone-remodeling responses. These effects vary according to metabolite identity, target-cell type, receptor signaling, and the local inflammatory context. This review summarizes current evidence linking gut microbiota dysbiosis, microbial metabolites, T helper 17/regulatory T-cell regulation, and osteoporotic fracture risk and repair. Microbiota-targeted approaches, including probiotics and prebiotics, together with engineered oral delivery and local immunoregulatory strategies, may provide opportunities to improve the osteoimmune environment. However, direct clinical evidence that these approaches prevent osteoporotic fractures or improve fracture healing remains limited. Direct evidence connecting gut microbiota-dependent T helper 17/regulatory T-cell responses with postmenopausal osteoporotic fracture healing is still scarce, and most conclusions are inferred from bone-loss models, standard fracture models, microbiota intervention studies, and bone regeneration models. Future studies should integrate microbiota profiling, barrier assessment, metabolite analysis, bone marrow and callus immune phenotyping, callus mineralization, and mechanical healing endpoints in osteoporotic fracture-specific models.

## 1 Introduction

As the global population ages, osteoporosis and osteoporotic fractures have become major public health challenges. Osteoporosis is characterized by reduced bone mass, deterioration of bone microarchitecture, and impaired bone strength, which together increase skeletal fragility and fracture susceptibility. Osteoporotic fractures represent its most severe clinical consequence. A Global Burden of Disease analysis reported that, in 2020, low bone mineral density (BMD) accounted for 17.2 million disability-adjusted life years and 477,000 deaths among individuals aged 40 years and older worldwide, with a rising mortality burden from 1990 to 2020 (1). Hip and vertebral fractures are particularly devastating in clinical practice. Cohort studies have shown that one-year mortality after osteoporotic hip fracture may approach one-third, and many survivors fail to regain their pre-fracture functional status (2–4). Notably, most fragility fractures occur in individuals with BMD T-scores above −2.5, indicating that BMD alone does not fully capture skeletal fragility or fracture risk (5).Successful fracture repair requires coordinated inflammatory responses, callus formation, mineralization, and remodeling, and disruption of these processes may compromise functional recovery. These clinical outcomes indicate that fracture prevention and treatment should not be restricted to increasing bone mineral density. Systemic bone fragility before fracture, immune resolution after fracture, callus formation, mineralization, remodeling, and mechanical healing quality are also central determinants of prognosis.

Postmenopausal osteoporosis (PMOP) is the most common form of primary osteoporosis. Traditional perspectives mainly emphasize the direct effects of estrogen deficiency on osteoblasts, osteoclasts, and the receptor activator of nuclear factor-κB ligand (RANKL)–receptor activator of nuclear factor-κB (RANK)–osteoprotegerin (OPG) axis. More recent advances in osteoimmunology have shown that immune cells and immune-derived mediators are active regulators of bone loss and repair (2, 3, 6). The gut microbiota is a major microbial ecosystem involved in host nutrient metabolism, mucosal barrier integrity, and immune homeostasis (7–9). Through the gut–bone axis, microbial communities may influence distant skeletal tissues by producing bioactive metabolites, maintaining epithelial barrier function, regulating lipopolysaccharide(LPS)-related inflammatory signaling, and shaping immune cell differentiation (8, 10). However, most human microbiome studies in osteoporosis are observational and do not establish gut dysbiosis as an independent cause of skeletal fragility or osteoporotic fracture. Dysbiosis may contribute to or amplify osteoimmune dysfunction, but it may also partly reflect the broader health status of affected individuals (11). Diet, physical activity, medication exposure, including antibiotics, aging, and comorbidities can influence microbial composition and skeletal health and should therefore be considered when interpreting microbiota–fracture associations. Metabolic conditions such as diabetes may further modify these relationships (12).

Clinical studies have reported differences in gut microbial diversity and composition between patients with PMOP and healthy postmenopausal women, and the abundance of specific taxa has been associated with bone mineral density, bone turnover markers, and inflammatory phenotypes (9, 13).

Among immune pathways involved in the gut–bone axis, the T helper 17 (Th17)/regulatory T (Treg) cell balance is particularly relevant to PMOP and osteoporotic fracture. Th17 cells can amplify osteoclastogenesis through interleukin-17 (IL-17), tumor necrosis factor-α (TNF-α), and RANKL-related signaling, whereas Treg cells restrain excessive inflammation and support immune resolution through interleukin-10 (IL-10), transforming growth factor-β (TGF-β), and cell-contact-dependent mechanisms (14–17). Estrogen deficiency, gut barrier impairment, microbial metabolite alterations, and LPS-mediated inflammatory activation may jointly disturb this balance, thereby linking gut dysbiosis to systemic osteoimmune remodeling in the osteoporotic state (14–16). Before fracture, Th17/Treg dysregulation tends to promote bone resorption, skeletal fragility, and increased fracture susceptibility. After fracture, however, the effects of this immune axis are stage- and context-dependent rather than uniformly detrimental or beneficial. During early repair, transient IL-17-producing T-cell responses may participate in the inflammatory and reparative responses required for callus formation. As healing progresses, appropriate resolution of inflammation and Treg-mediated immunoregulation become increasingly important for the transition toward tissue repair and remodeling, whereas persistent or excessive Th17/IL-17 activity in a pro-osteoclastogenic inflammatory environment may interfere with this transition. Thus, the effects of the Th17/Treg axis during fracture healing depend on the timing, cellular source, magnitude, and local inflammatory context of the response (18–20). However, direct evidence linking gut microbiota-driven Th17/Treg responses to osteoporotic fracture healing remains limited. Notably, direct evidence that gut-derived Th17 cells traffic to the fracture callus currently comes from a single murine fracture study, and comparable evidence demonstrating this gut-to-callus trafficking pathway in humans has not yet been established.Many current conclusions are still inferred from PMOP models, general fracture models, inflammatory bone loss models, or bone defect studies, and therefore require further validation in osteoporotic fracture-specific models. This proposed gut microbiota–immune–callus axis is summarized in **Figure 1**, which illustrates how PMOP-associated dysbiosis, barrier dysfunction, systemic inflammatory activation, Th17/Treg dysregulation, and intestinal immune-cell trafficking may converge on the fracture callus microenvironment.

**Figure 1:**
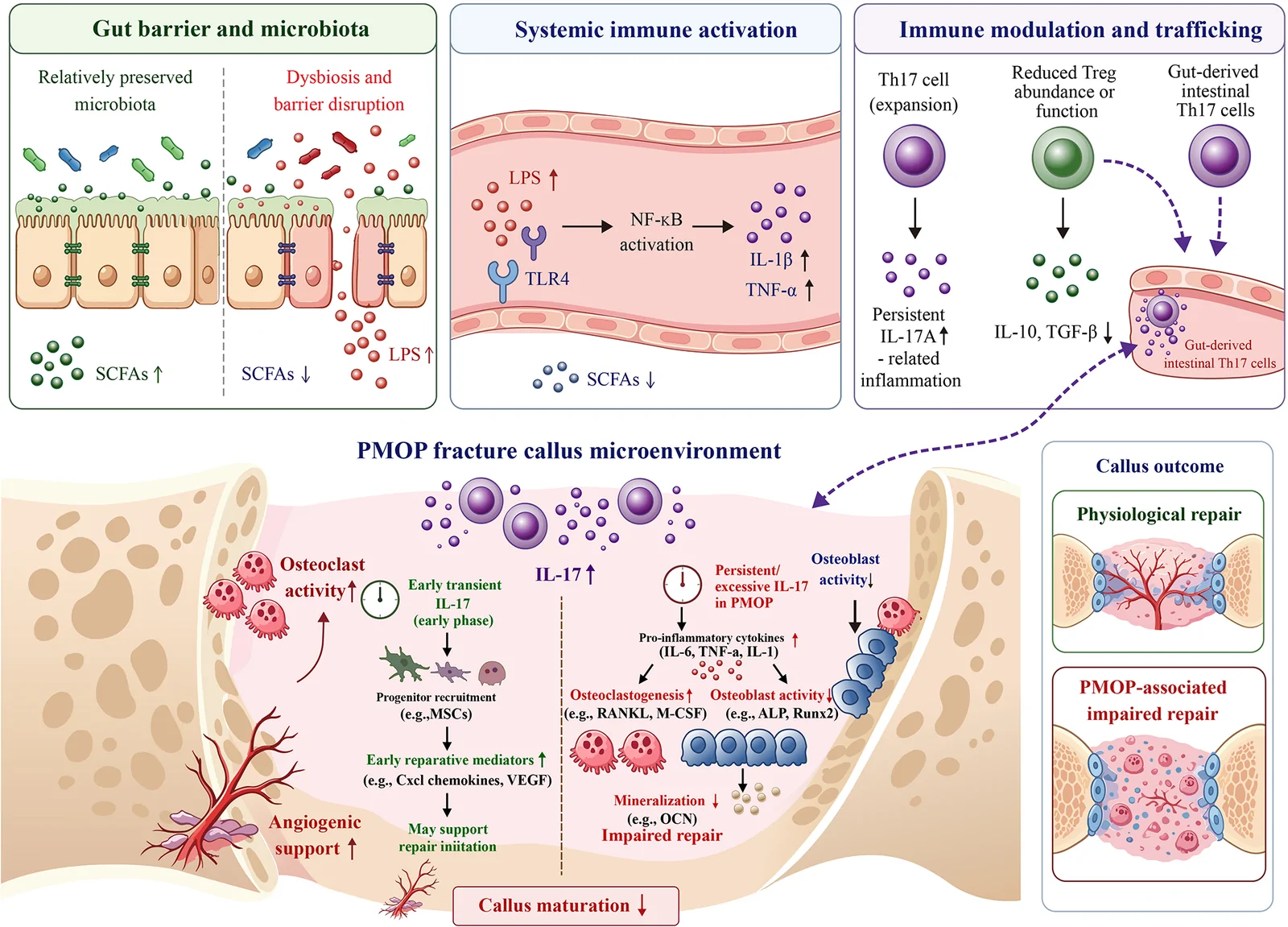
Proposed mechanism linking gut dysbiosis to impaired fracture repair in postmenopausal osteoporosis. In the relatively preserved state, the gut microbiota supports barrier integrity and maintains higher levels of short-chain fatty acids (SCFAs). In PMOP-associated dysbiosis, barrier disruption is accompanied by reduced SCFAs and increased lipopolysaccharide (LPS) translocation, leading to TLR4/NF-κB-mediated systemic immune activation with increased pro-inflammatory cytokines. These changes are associated with Th17 expansion, Treg reduction, and trafficking of gut-derived intestinal Th17 cells, which together reshape the fracture callus microenvironment. Within the callus, the effects of IL-17 are stage- and context-dependent. Transient IL-17 signaling during the early phase may participate in early inflammatory and reparative responses, whereas persistent or excessive IL-17 signaling in the PMOP inflammatory milieu may sustain pro-inflammatory and osteoclastogenic activity, reduce osteogenic support, impair mineralization, and delay callus maturation, ultimately contributing to compromised repair outcomes.

Previous reviews have discussed the gut–bone axis in osteoporosis from the perspectives of dysbiosis, microbial metabolites, immune regulation, endocrine interactions, and biomaterial-based intervention (12, 14, 21). However, the connection between these mechanisms and the continuum from osteoporotic fracture susceptibility to stage-specific immune regulation of fracture repair has received less integrated attention. This review therefore focuses on the gut microbiota–Th17/Treg axis across the progression from postmenopausal skeletal fragility to fracture repair. Particular attention is given to evidence linking intestinal barrier dysfunction, microbial metabolites, systemic and bone-marrow immune remodeling, and immune-cell trafficking with fracture-related outcomes, while distinguishing findings derived from human studies, osteoporosis models, conventional fracture models, and osteoporotic fracture-specific evidence.

## 2 Postmenopausal gut microbiota dysbiosis and risk of osteoporotic fracture

### 2.1 Alterations in the microbiota and disruption of Th17/Treg homeostasis in postmenopausal osteoporosis

Gut microbiota alterations in PMOP are increasingly linked to a low-grade inflammatory and osteoimmune phenotype. In patients with PMOP, fecal bacterial richness is reduced and β-diversity differs from that of individuals with normal bone mass. At the phylum level, this dysbiosis is characterized mainly by reduced Firmicutes and increased Campylobacterota, with additional changes in Bacteroidota and Proteobacteria. These microbial changes are accompanied by elevated circulating lipopolysaccharide (LPS), reduced regulatory T cells (Treg) and interleukin-10 (IL-10), and increased T helper 17 cells (Th17), IL-17A, IL-1β, IL-6, and tumor necrosis factor-α (TNF-α) (13). Together, these findings suggest that PMOP-related dysbiosis is associated with enhanced gut-derived inflammatory stimulation and weakened peripheral immune regulation. In another cohort of postmenopausal women, integrated 16S rRNA sequencing and LC-MS-based metabolomics showed that reduced BMD and altered bone metabolic indexes were associated with changes in gut microbial composition and fecal metabolic phenotypes (9). Mechanistic studies in sex steroid deficiency models provide further support for this microbiota–immune–bone connection. Under estrogen-deficient conditions, increased gut permeability enhances microbiota-driven inflammatory signaling, expands Th17 cells in the small intestine and bone marrow, and upregulates osteoclastogenic mediators, including TNF, receptor activator of nuclear factor κB ligand (RANKL), and IL-17 (15). Conversely, in germ-free mice, sex steroid deficiency fails to induce comparable osteoclastogenic cytokine production, bone resorption, or trabecular bone loss, indicating that the microbiota is required for the full inflammatory bone-loss response to estrogen deficiency (15). In OVX rats, Lactobacillus rhamnosus GG (LGG) improves bone microarchitecture and biomechanical properties, modulates CTX-I, P1NP, calcium, and RANKL levels, and increases GLP-2R and tight junction protein expression, consistent with improved intestinal barrier integrity (16). LGG also partially reverses OVX-induced Th17 expansion, bone marrow IL-17 and TNF-α elevation, and the reduction of Treg-related TGF-β and IL-10, supporting its role in restoring gut and bone marrow immune balance (16). These findings suggest that, at least in this OVX model, the bone-protective effect of LGG is mediated not only by local skeletal effects but also by barrier improvement and restoration of immune homeostasis.In a small retrospective study, patients with PMOP showed reduced abundance of Subdoligranulum, Norank_f_Muribaculaceae, and Alistipes, together with lower fecal propionate levels, and these changes were associated with lumbar spine or femoral neck BMD (17). Subdoligranulum was also associated with acetate and butyrate levels, suggesting that reduced SCFA output may be one metabolic feature linking dysbiosis to impaired anti-inflammatory regulation (17). In an OVX model, combined intervention with *Butyricicoccus pullicaecorum* and the tryptophan metabolite 3-hydroxyanthranilic acid (3-HAA) was reported to improve femoral pathological changes, attenuate Th17/Treg dysregulation, and remodel microbial pathways related to nutrient metabolism and immune function (22). Overall, PMOP-associated dysbiosis may shape a pro-osteoclastogenic immune environment before fracture through gut barrier impairment, LPS-related inflammation, insufficient SCFA metabolism, and Th17/Treg dysregulation. This pre-existing immune state may increase skeletal fragility and leave subsequent fracture repair to begin under less favorable osteoimmune conditions.

### 2.2 Alterations in the microbiota and the risk of decreased BMD and osteoporotic fractures

Early evidence linking microbiota alterations to skeletal fragility was mainly based on BMD and bone turnover-related indices, whereas recent studies have extended this association beyond bone density alone. Early 16S rRNA sequencing studies showed that patients with osteoporosis or osteopenia had gut microbial β-diversity profiles distinct from those of individuals with normal bone mass, with differences in taxa such as Blautia, Parabacteroides, and members of Ruminococcaceae (23). In a Chinese elderly cohort, individuals with low BMD had lower OTU counts and reduced bacterial abundance at several taxonomic levels than controls. Lachnospiraceae, Roseburia, Bifidobacterium, and Lactobacillus were positively correlated with BMD or T-score, whereas LPS biosynthesis-related functional pathways were enriched in participants with low BMD (24). In postmenopausal women, combined 16S rRNA sequencing and LC-MS metabolomics extended this association to the metabolic level. Patients with PMOP showed reduced microbial richness and diversity, together with altered fecal metabolites, lumbar spine BMD, and bone turnover markers such as P1NP and CTX-I (9). These findings indicate that microbiota-related signatures may reflect a composite phenotype involving bone mass, bone turnover activity, and host metabolic status, rather than changes in a single taxon alone.

Evidence related to fracture risk further extends this interpretation. In Japanese postmenopausal women, Bacteroides abundance was higher in individuals with higher vitamin K2 levels, and lower Bacteroides abundance was associated with a higher prevalence of previous fractures. Rikenellaceae was enriched in participants with low BMD and high TRACP-5b, suggesting that microbial features may be linked to vitamin metabolism, bone resorption activity, and fracture history (25). However, these microbial associations are not consistent across populations. The inconsistent direction of Bacteroides across studies suggests that microbiota-based biomarkers should be interpreted in relation to dietary patterns, vitamin metabolism, population background, and disease stage, rather than as isolated diagnostic indicators (24, 25). Animal studies further suggest that microbiota alterations may affect bone quality beyond BMD. Long-term antibiotic-induced microbiota disruption reduces femoral bending strength and bone tissue material properties in mice, and this mechanical decline cannot be fully explained by changes in bone geometry, suggesting that microbiota status may affect components of bone strength beyond BMD (26). In OVX osteoporotic rats, intermittent PTH treatment is accompanied by changes in microbial complexity, community structure, and predicted functions related to butyrate metabolism. These findings suggest that anti-osteoporotic treatment may be accompanied by shifts in intestinal metabolic function, although whether these changes contribute to the skeletal response requires further validation (27). A prospective cohort study further linked microbiota characteristics to incident fracture as a clinical endpoint. In the FINRISK cohort, fecal metagenomic sequencing was performed in 7,043 participants, among whom 1,092 incident fracture events were recorded during a median follow-up of 18 years. Higher Shannon α-diversity was associated with lower fracture risk, and principal components of β-diversity were also related to fracture risk. At the phylum level, increased Proteobacteria abundance was associated with higher fracture risk, whereas increased Tenericutes abundance was associated with lower fracture risk (28). Functional analysis further suggested that amino acid metabolism, LPS biosynthesis, and butyrate synthesis-related pathways may participate in the association between microbiota and fracture risk (28). Because baseline BMD was not available in this cohort, these results support a temporal association between microbiota and incident fracture risk but do not determine whether this relationship is independent of bone mass.

A recent genetic prediction study reported that genetically predicted Eubacterium xylanophilum group and Enterobacteriaceae were associated with increased fracture risk, whereas Sellimonas, Ruminococcus2, Escherichia-Shigella, and Veillonella showed inverse associations (29). The study further suggested that HDL-related metabolites may partially mediate the microbiome-fracture association.Lipid metabolite analyses have also shown that several HDL-related metabolites, 3-hydroxybutyric acid, and sphingomyelin are associated with osteoporotic fracture risk, providing metabolic clues that may help interpret the relationship between host metabolism and fracture susceptibility (30). Existing evidence suggests that dysbiosis does not correspond to a single bone metabolic indicator, but may contribute to a composite risk profile for skeletal fragility together with increased bone turnover, inflammatory burden, metabolic dysfunction, and reduced bone strength.

## 3 Th17/Treg dynamics and stage-dependent IL-17 signaling in osteoporotic fracture repair

### 3.1 Th17/Treg regulation during the early inflammatory response after osteoporotic fracture

Osteoporotic fracture repair begins in a host environment already characterized by skeletal fragility and immune dysregulation. Patients with PMOP have been reported to exhibit gut microbiota dysbiosis, LPS-associated inflammatory input, enhanced Th17 activity, and reduced Treg regulation. In postmenopausal women with low BMD, increased IL-17 levels and an elevated Th17/Treg ratio are also associated with bone loss (13, 31). These findings suggest that the immune response after osteoporotic fracture may occur within a pre-existing pro-inflammatory and pro-osteoclastogenic microenvironment. After fracture, a transient inflammatory response is required to initiate repair. Fracture hematoma rapidly recruits neutrophils, macrophages, T cells, and other immune cells to the injury site. TNF-α, IL-1β, IL-6, and multiple chemokines participate in necrotic tissue clearance, angiogenesis, mesenchymal progenitor recruitment, and early cartilaginous callus formation (18, 32). In contrast, persistent inflammation can shift the local microenvironment from damage clearance toward tissue destruction, thereby interfering with cartilaginous callus formation, callus mineralization, and subsequent remodeling (18, 33). Th17 cells, γδT cells, and IL-17 signaling may amplify the early inflammatory response after fracture and participate in mesenchymal progenitor activation, osteoblast differentiation, and initiation of bone repair (32, 33). This response may favor damage clearance and callus formation in the short term. However, in an osteoporotic and dysbiotic background with persistent LPS, TNF, IL-6, and RANKL signaling, sustained Th17/IL-17 activity may favor an osteoclastogenic inflammatory profile, prolonging the inflammatory phase and increasing bone resorption pressure (13, 15). In contrast, Tregs not only restrict excessive inflammatory responses but also directly participate in fracture repair. After fracture, Tregs can accumulate in the callus through recruitment from circulation, peripheral induction, and local expansion. Callus Tregs highly express amphiregulin and promote osteoprogenitor proliferation and differentiation through PI3K/AKT signaling, whereas Treg reduction impairs fracture repair (34). CCR8+ Tregs can also infiltrate the injury site in response to CCL1 signaling and promote skeletal stem cell accumulation, osteogenic differentiation, and bone repair through BATF-dependent regulation of *Grn* expression and PGRN secretion (19). Thus, early fracture healing depends less on unidirectional suppression of Th17 responses or enhancement of Tregs than on a controlled transition from inflammatory initiation to timely resolution and osteogenic repair. Under PMOP-associated dysbiosis, LPS-related inflammatory stimulation, and estrogen deficiency, Th17/IL-17 responses may be more likely to persist, whereas the regulatory and osteogenic repair-promoting functions of Tregs may be weakened. This dysregulation may impair the transition from inflammation to cartilaginous callus formation, mineralization, and remodeling.

### 3.2 Cellular sources of IL-17 and stage-dependent effects of microbiota-associated Th17 responses

IL-17 is an important effector signal connecting gut microbiota-associated Th17 responses with fracture repair. Local γδT cells in the fracture callus can rapidly produce IL-17A, and microbiota-induced intestinal Th17 cells have also been shown to migrate into the callus and participate in the local immune response (20, 35). γδT cells are among the major early sources of IL-17 in the fracture microenvironment. As tissue-resident lymphocytes enriched at barrier sites, γδT cells can rapidly release effector cytokines after injury with limited dependence on classical antigen presentation. The gut contains distinct γδT-cell populations in the epithelial layer, lamina propria, and Peyer’s patches. Lamina propria γδT cells can produce IL-17 and IL-22 and are regulated by local microbial and epithelial signals (36). Supportive evidence from chronic stress models suggests that gut microbial changes can influence intestinal γδ17 responses, as reduced Lactobacillus johnsonii is associated with enhanced colonic γδ17 differentiation (37). These findings indicate that gut microbiota can shape intestinal IL-17-producing cell responses, although their contribution to fracture repair depends on cellular source and disease context. Different cellular sources of IL-17 may have distinct roles during fracture repair. In fracture models, callus γδT cells appear to be driven primarily by local injury and rapidly expand, producing IL-17 during the early inflammatory and reparative response. In parallel, the microbiota contributes an intestinal Th17-cell component that can traffic to the callus and participate in local immunity (20). The effect of IL-17 on fracture repair therefore depends on its cellular source, target cells, and inflammatory stage.

IL-17 signaling can contribute to the initiation of early fracture repair.Bone injury and fracture-related models have shown that deficiency of IL-17A impairs new bone formation, while IL-17A derived from γδT cells promotes the proliferation and osteogenic differentiation of mesenchymal progenitor cells, thereby enhancing bone regeneration (35). *In vitro* studies have shown that activated T cells and their conditioned media promote osteogenic differentiation of human MSCs. Soluble factors associated with Th17 cells exert a strong pro-osteogenic effect, IL-17A and IL-17F promote MSC osteogenic maturation, and IL-17A can synergize with BMP2 (38). Studies by Zhang et al. further revealed that IL-17F can upregulate osteogenic factors such as Runx2, Osterix, and BMP2, and promote proliferation, differentiation, and mineralization of osteoblasts through MAPK/ERK1/2 signaling (39). These results indicate that IL-17 family signaling can support progenitor cell activation and osteogenic programs at specific stages and in specific cellular contexts, providing early inflammatory and osteogenic coupling signals for callus formation.

IL-17, however, is not uniformly pro-osteogenic. Conditional knockout studies of IL-17RA have shown that deletion of IL-17RA in Prx1+ mesenchymal progenitor cells alters the cartilage and bone composition of the early fracture callus, indicating that IL-17 receptor signaling participates in the regulation of callus cellular composition (40). *In vitro* experiments show that IL-17A can promote fracture-activated periosteal cell migration but inhibits their osteogenic differentiation (40). These findings suggest that IL-17 is not a unidirectional osteogenic signal, but a context-dependent regulator of cell mobilization and repair progression after injury. During the early fracture phase, IL-17 may support cell recruitment and local inflammatory amplification. Thereafter, inflammatory signaling needs to decline so that osteogenic differentiation, matrix deposition, and mineralization can become dominant. If IL-17 signaling remains elevated for a prolonged period, it may sustain inflammation and osteoclast activation, thereby weakening its early pro-repair effects and impairing bone healing.

This dual effect indicates that IL-17 signaling in bone repair is stage-dependent, and this stage specificity may become more pronounced in the pro-inflammatory immune background of PMOP. Th17 cells are recognized as a pro-osteoclastogenic helper T-cell subset that links T-cell activation to inflammatory bone destruction by amplifying IL-17-related inflammation, upregulating RANKL expression, and promoting osteoclastogenesis (41). In estrogen-deficiency models, elevated levels of Th17 cells and IL-17 are associated with trabecular bone loss, and IL-17 neutralization can reduce bone loss and ameliorate aberrant osteoclastogenesis and osteogenic differentiation (42). In the context of PMOP-related dysbiosis, increased LPS exposure, and insufficient Treg regulation, microbiota-associated Th17/IL-17 signaling may be more likely to sustain a pro-inflammatory and pro-osteoclastogenic response rather than remain confined to early repair initiation (13, 15). Thus, in osteoporotic fracture repair, the biological effects of IL-17 depend on both source and duration. Locally produced IL-17 in the early fracture phase may help initiate repair, whereas sustained IL-17 release from microbiota-associated Th17 cells could maintain inflammation, promote bone resorption, and interfere with bone formation. This dysregulation may become more evident when Treg-mediated immune regulation is insufficient, potentially affecting callus remodeling and fracture healing.

### 3.3 Gut-derived Th17 cells enter the callus and participate in fracture repair

Increasing evidence indicates that the gut microbiota can shape intestinal Th17 responses that subsequently influence bone-associated immune environments. Specific microbial taxa can drive intestinal Th17 differentiation. For example, segmented filamentous bacteria (SFB) induce CD4+ T cells in the intestinal lamina propria to produce IL-17 and IL-22, making the gut a critical site for microbiota-dependent Th17 responses (43). This response is not confined to the local intestinal mucosa. SFB-specific Th17 cells are first activated in gut-draining mesenteric lymph nodes, where IL-6 promotes early RORγt expression.When IL-6 is insufficient, IL-21 and IL-23 can compensate, after which these cells home to the intestinal lamina propria in an integrin β7-dependent manner (44). This process links microbial antigen recognition, Th17 differentiation, and homing to the intestinal lamina propria, providing a cellular basis for microbiota-dependent Th17 responses. A recent immune migration atlas further showed that colon-derived immune cells can exit the intestine through S1P receptor-related signaling and acquire different fates depending on the distal inflammatory tissue. This migrating population includes RORγ+ cells, and antibiotic treatment reduces the migration of colon-derived immune cells to the spleen and lymph nodes (45). These findings support the plausibility that gut-derived immune cells can contribute to distal inflammatory sites, including bone-associated tissues, under appropriate injury or inflammatory conditions.

Bone loss models further indicate that gut-derived Th17 cells can exit the intestinal compartment and exert different effects depending on the bone tissue environment.In OVX and sustained PTH-associated bone loss models, microbiota-dependent intestinal TNF+ T cells and Th17 cells can egress via S1PR1-mediated pathways and enter the bone marrow, coupling with TNF, IL-17, and RANKL-related pro-osteoclastogenic signals to promote bone resorption and trabecular bone loss (46, 47). These findings indicate that gut-derived Th17 cells can leave the intestinal immune compartment and enter bone-associated tissues. Femoral fracture models provide more direct evidence that gut-derived Th17 cells participate in callus repair. After fracture, callus γδT cells undergo rapid expansion and produce IL-17A, indicating that the local fracture site itself is capable of initiating an early IL-17-mediated immune response, whereas recruited gut-derived Th17 cells later supplement the local IL-17 response and contribute to callus repair (20). More specifically, microbiota-induced gut-derived Th17 cells can enter the callus and participate in the local IL-17 response through S1PR1-dependent egress and CCL20-mediated recruitment (20). These findings indicate that the gut microbiota influences fracture repair not only through systemic inflammation, but also by inducing migratory Th17 cells that enter the callus and directly participate in the local immune environment at the fracture site.

Although gut-derived Th17 cells can migrate to bone-associated tissues, their biological effects vary according to the local tissue microenvironment and disease context. Upon entry into the bone marrow, gut-derived Th17 cells encounter an environment of sustained enhanced bone resorption and elevated osteoclast activity, making them more likely to interact with TNF, IL-17, and RANKL signaling, thereby promoting bone resorption (46, 47). In a standard fracture model, Th17 cells entering the callus during the early inflammatory phase can supplement local IL-17 signaling and participate in inflammatory initiation, progenitor cell recruitment, and callus formation (20). Thus, the divergent effects of gut-derived Th17 cells in bone loss and fracture repair are shaped by local tissue environment, inflammatory duration, Treg-mediated regulation, and the stage of osteogenic repair. In osteoporotic fractures, PMOP-associated dysbiosis, barrier dysfunction, and LPS-related inflammatory stimulation may expand or maintain intestinal Th17 responses, while estrogen deficiency further facilitates coupling between Th17/IL-17 signaling and pro-osteoclastogenic mediators such as TNF and RANKL (13, 15). If Treg-mediated anti-inflammatory and osteogenic support is insufficient, the Th17 response within the callus may have difficulty transitioning from reparative inflammation to inflammation resolution in a timely manner. Under these conditions, gut-derived Th17 cells may shift from short-term participants in callus formation to sustained contributors to inflammation, osteoclast differentiation, and impaired callus mineralization and remodeling.

### 3.4 Th17/Treg dysregulation and impaired callus repair in the PMOP context

PMOP-related fracture repair occurs in a host environment characterized by reduced bone mass, heightened inflammatory tone, and impaired immune homeostasis (13). In postmenopausal women with low BMD, increased Th17 frequency, elevated IL-17 levels, and a higher Th17/Treg ratio are associated with lower lumbar spine and femoral neck T-scores (31). Recent population evidence further shows that serum IL-17 levels are higher in postmenopausal women with osteoporosis than in non-osteoporotic controls. IL-17 is negatively correlated with DXA-derived BMD or T-scores and estradiol, and positively correlated with FRAX-estimated 10-year risks of major osteoporotic and hip fractures (48).Together, these findings indicate that postmenopausal bone loss is not limited to abnormal bone cell remodeling, but is frequently accompanied by enhanced Th17/IL-17 activity and insufficient Treg-mediated regulation.

Animal models provide mechanistic support for this clinical association. Through microbiota-dependent mechanisms, sex hormone deficiency increases intestinal permeability, expands Th17 cells, and upregulates TNF, RANKL, and IL-17 in the small intestine and bone marrow (15). In estrogen deficiency models, elevations in Th17 cells and IL-17 levels are associated with trabecular bone loss. IL-17 neutralization can alleviate bone loss and improve abnormal osteoclastogenesis and osteogenic differentiation (42). In inflammatory bone destruction, Th17 cells also promote bone resorption through IL-17-mediated inflammatory amplification, RANKL expression, and osteoclastogenesis (41). The effects of IL-17 on bone repair and resorption are stage-dependent. Early local IL-17 signaling may participate in injury clearance, progenitor cell recruitment, and callus formation, whereas persistent Th17/IL-17 signaling under estrogen deficiency, LPS-related inflammatory stimulation, and sustained TNF/RANKL activity may favor osteoclastogenic inflammation. These divergent outcomes depend on IL-17 source, inflammatory duration, local reparative context, and Treg-mediated regulation.

Tregs, in contrast, play a dual role in fracture repair by suppressing excessive inflammation and promoting bone tissue regeneration. Bone injury studies have shown that CCR8+ Tregs enter the injury site in response to CCL1 signaling and promote skeletal stem cell accumulation, osteogenic differentiation, and bone repair through BATF-mediated regulation of *Grn* expression and PGRN secretion (19). In standard fracture models, callus Tregs accumulate within injured bone tissue through recruitment from circulation, peripheral induction, and local expansion. These cells highly express amphiregulin and promote osteoprogenitor proliferation and differentiation. Treg depletion during fracture healing impairs callus formation, delays bone repair, and predisposes to impaired union (34). If Treg abundance, activity, or reparative function is insufficient in the PMOP state, ongoing inflammation may be less effectively restrained, and skeletal stem cell accumulation, osteoprogenitor proliferation, and new bone formation may be weakened.

This functional divergence between Th17 cells and Tregs may help explain why osteoporotic fracture healing is prone to delayed or impaired repair. In standard fracture models, callus γδT cells and microbiota-induced gut-derived Th17 cells participate in the early IL-17-related repair response, with Th17 cells entering the callus to promote progenitor cell recruitment and callus formation (20). Under PMOP-associated dysbiosis, LPS-related inflammatory stimulation, and estrogen deficiency, sustained Th17/IL-17 signaling may instead couple with TNF and RANKL networks and prolong the inflammatory phase when Treg-mediated regulation is insufficient. Under these conditions, the fracture site may fail to transition efficiently from the inflammatory phase toward resolution, cartilaginous callus formation, mineralization, and remodeling. Together, these factors may result in prolonged local inflammation, reduced bone formation, impaired remodeling, insufficient callus formation, delayed mineralization, and reduced post-healing mechanical performance. **Figure 2** summarizes the source-dependent and stage-dependent role of IL-17 signaling in osteoporotic fracture repair, highlighting the contrast between early local IL-17-mediated repair initiation and persistent PMOP-associated Th17/IL-17 inflammation that may impair callus maturation when Treg-mediated regulation is insufficient.

**Figure 2:**
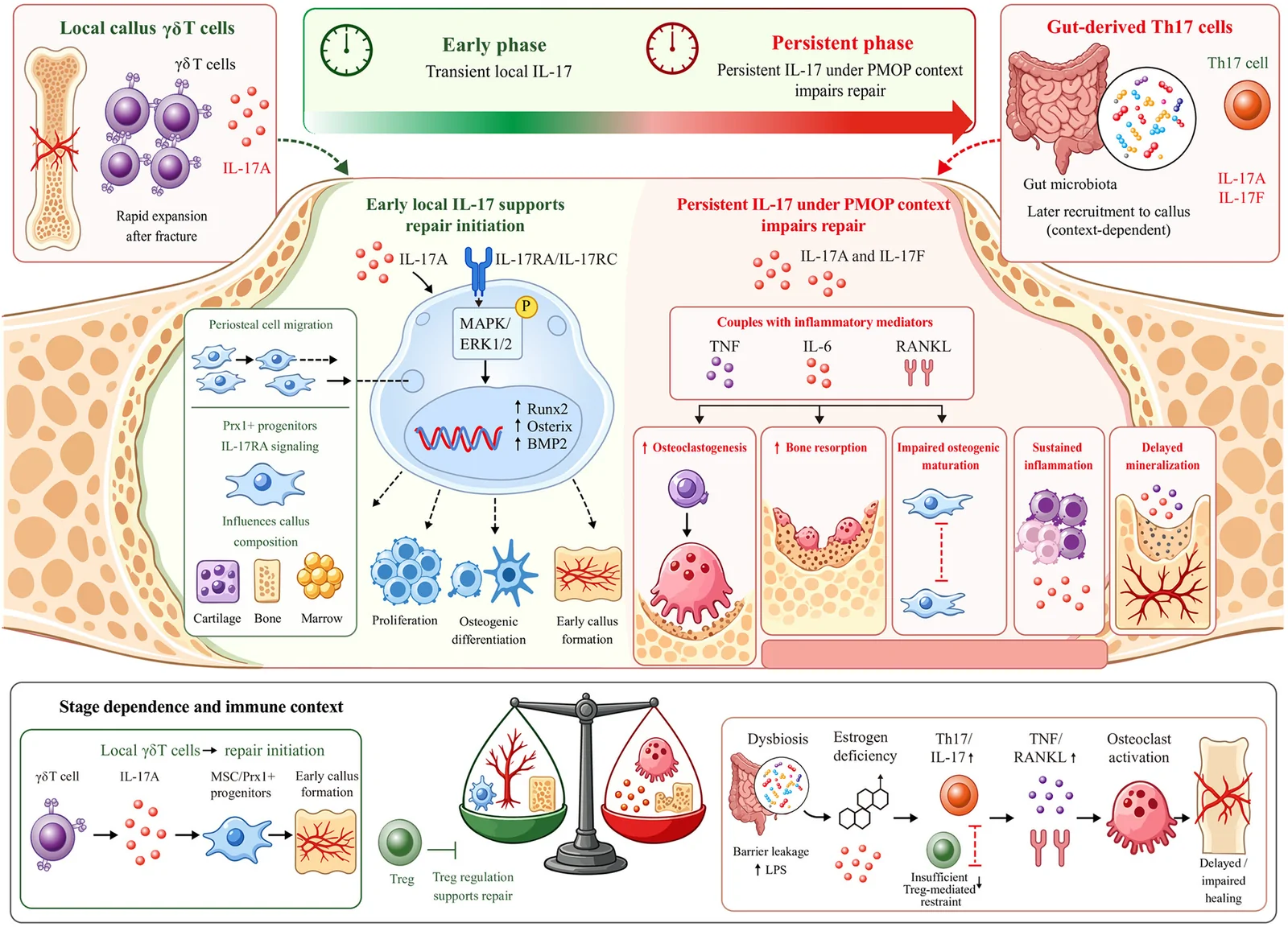
Source-dependent and stage-dependent effects of IL-17 signaling in osteoporotic fracture repair. The mechanistic framework depicted in this image is based predominantly on murine and other preclinical studies, and direct validation in human osteoporotic fracture callus remains limited. In the early phase after fracture, local callus γδ T cells rapidly expand and produce IL-17A, which may support repair initiation by promoting periosteal cell migration, Prx1+ progenitor cell responses, osteogenic signaling, and early callus formation. IL-17F can activate MAPK/ERK1/2-related osteogenic programs in osteoblasts, whereas IL-17RA signaling in Prx1+ mesenchymal cells influences early callus composition. However, under a PMOP-associated inflammatory background, persistent IL-17 signaling may couple with TNF, IL-6, and RANKL, thereby sustaining inflammation, enhancing osteoclastogenesis, increasing bone resorption, impairing osteogenic maturation, delaying mineralization, and compromising repair. Gut microbiota-associated Th17 cells may be recruited to the callus in a context-dependent manner and contribute to local IL-17 responses. Treg-mediated regulation helps restrain excessive inflammation and supports repair, whereas insufficient Treg control may shift the balance toward delayed or impaired healing.

## 4 Intestinal barrier dysfunction and remodeling of the bone marrow immune microenvironment

### 4.1 Intestinal barrier dysfunction and bone immune dysregulation

When intestinal barrier integrity is compromised, bacterial products in the intestinal lumen can more readily cross the epithelial barrier and transmit the effects of dysbiosis to extraintestinal immune compartments, including the bone marrow. In patients with PMOP, alterations in the microbiota are often accompanied by increased endotoxin exposure, elevated pro-inflammatory mediators, and weakened anti-inflammatory regulation (13). When this microbial state is accompanied by increased intestinal permeability, LPS and other microbe-associated products may reach extraintestinal immune cells and enhance TNF-α, IL-17, and RANKL related pro-osteoclastogenic responses in the bone marrow. In sex hormone deficiency models, increased intestinal permeability occurs together with elevated TNF, RANKL, and IL-17 in the small intestine and bone marrow, as well as trabecular bone loss (15). In contrast, germ-free mice do not develop comparable osteoclastogenic cytokine production, bone resorption, or trabecular bone loss after sex hormone deficiency (15). In the same model, LGG reduce intestinal permeability, increase tight junction-related protein expression, dampen intestinal and bone marrow inflammation, and protect against bone loss (15).

In patients with PMOP, elevated endotoxin levels alone do not prove structural mucosal damage. Barrier impairment still requires support from permeability testing, tight junction markers, and histological evidence (49). After transplantation of fecal microbiota from PMOP donors into antibiotic-treated mice, recipient animals showed bone loss, intestinal mucosal injury, decreased expression of claudin proteins and ZO-1, increased FITC-dextran absorption, and elevated soluble CD14. Serum and bone tissue levels of TNF-α and IL-17A were also increased (49). These findings indicate that PMOP-associated microbiota can transmit a phenotype characterized by reduced barrier integrity, inflammatory activation, and bone loss. Because this study focused on bone mass and inflammation rather than callus formation or fracture healing, it mainly supports a role for PMOP-associated microbiota in bone loss through barrier disruption and immune activation. Its effects on osteoporotic fracture repair still require validation in dedicated fracture models. Microbial metabolites also contribute to barrier regulation (50, 51). Loss of butyrate-producing bacteria or insufficient SCFA production may weaken epithelial tight junctions, facilitate the entry of microbe-associated products into the circulation, and aggravate low-grade inflammation and osteoclast activation (52).

Intervention studies that restore barrier function provide complementary support for this mechanism (53). In OVX rats, LGG attenuates intestinal mucosal injury, increases tight junction-associated proteins, reduces TNF-α and IL-17 in the colon and bone marrow, and increases IL-10 and TGF-β, accompanied by improved bone microarchitecture and biomechanical properties (16). Supplementation with *Parabacteroides distasonis* increases ZO-1 and occludin expression and decreases serum LPS. Th17/Treg-related markers and cytokine profiles in mesenteric lymph nodes also shift toward a less inflammatory state, accompanied by improved distal femoral trabecular microarchitecture and bending strength (54). *Lactobacillus acidophilus* also increases Tregs, decreases Th17 cells, reduces osteoclastogenic factors such as RANKL and IL-17, and improves bone mass and trabecular structure in OVX mice (55). In a post-antibiotic dysbiosis model, aberrant microbiota reconstruction increases intestinal permeability and reduces trabecular bone mass, whereas mucosal barrier protection or *Lactobacillus reuteri* intervention mitigates both barrier disruption and bone loss (56).

### 4.2 LPS-related inflammatory signaling and Th17/Treg dysregulation

When intestinal permeability increases, circulating LPS can contribute to skeletal immune dysregulation by activating innate immune responses and reshaping the bone marrow inflammatory microenvironment (57). These factors further alter the conditions for CD4+ T cell differentiation. In a low-inflammatory environment, TGF-β favors Foxp3+ Treg generation. When IL-6 is elevated, it inhibits TGF-β-induced Foxp3 expression and, together with TGF-β, promotes IL-17-producing T-cell differentiation (58). Supportive *in vitro* evidence shows that E. coli LPS stimulation of peripheral T cells activates TLR4/NF-κB signaling, increases IL-6 and IL-17 expression, and raises the Th17 proportion, Th17/Treg ratio, and RORγt/Foxp3 ratio (59). Thus, LPS-TLR4/NF-κB activation can promote a cytokine environment that favors Th17 differentiation over Treg regulation, shifting the immune response toward a Th17-dominant inflammatory state.

LPS-associated Th17/Treg dysregulation is closely linked to TNF-α, IL-17, and RANKL signaling, which together support osteoclast activation and bone loss (60). The improvement of BMD, bone strength, bone microarchitecture, and Breg-mediated anti-osteoclastogenic regulation after *Bacillus coagulans* intervention further supports the involvement of the gut-immune-bone axis (60).Mechanistically, estrogen deficiency increases intestinal permeability and endotoxin exposure and elevates TNF-α, RANKL, and IL-17 in the small intestine and bone marrow (15). In germ-free mice, sex hormone deficiency fails to induce comparable osteoclastogenic cytokine production and trabecular bone loss, suggesting that microbiota-associated inflammatory signaling is required for the full pro-osteoclastogenic bone marrow response to estrogen deficiency (15).

LPS-associated signaling can also enhance bone resorption at the level of osteoclast differentiation. *In vitro* studies have shown that LPS alone does not induce RAW264.7 cells to mature into osteoclasts,however, after RANKL pretreatment, LPS can promote osteoclastogenesis via TLR4-dependent TNF-α signaling. Both TLR4 inhibitors such as TAK-242 and TNF-α antibodies can inhibit this process, suggesting that LPS is more likely to enhance osteoclast formation under conditions where RANKL or inflammatory priming already exists (57). In states of estrogen deficiency, levels of RANKL, TNF-α and IL-17 are already elevated. When gut-derived LPS exposure is present simultaneously, the bone marrow pro-osteoclastogenic network may be more prone to sustained activation. Early studies found that IL-17 secreted by Th17 cells can promote RANKL expression and enhance osteoclastogenesis, thereby driving bone resorption (41). Under estrogen deficiency and chronic low-grade inflammation, Th17 expansion and elevated IL-17 may further amplify LPS-associated inflammatory effects and bone loss. In estrogen-deficient states, increased intestinal permeability, elevated endotoxin exposure, and enhanced TNF-α, IL-17, and RANKL expression in the bone marrow may converge to sustain a pro-osteoclastogenic inflammatory environment. After fracture, this inflammatory milieu may affect local immune cells and bone metabolic cells. However, current evidence mainly supports the involvement of LPS-associated inflammation in PMOP bone loss and fragility. Evidence from PMOP fracture models is still needed to determine whether LPS-driven Th17/Treg dysregulation directly alters callus formation, bone remodeling, or healing quality.

### 4.3 Intestinal Th17 recruitment and bone marrow pro-osteoclastogenic microenvironment

Once intestinal T cells enter the bone marrow, microbiota-associated immune responses are no longer confined to the intestinal mucosa and may contribute to a local inflammatory milieu that favors osteoclastogenesis. Under estrogen-deficient conditions, intestinal TNF^+^ T cells and Th17 cells expand in a microbiota-dependent manner (46). S1PR1 mediates the egress of these T cells from gut-associated lymphoid tissue, while CXCR3-related signaling facilitates the entry of TNF^+^ T cells into the bone marrow. Once in the bone marrow, TNF^+^ T cells induce CCL20 expression in bone marrow cells via TNF, and CCL20 further recruits CCR6^+^ Th17 cells. Blocking intestinal T cell egress or inhibiting CCL20-mediated recruitment reduces the accumulation of TNF^+^ T cells and Th17 cells in the bone marrow and alleviates OVX-induced trabecular bone loss (46). In this cascade, TNF^+^ T cells promote the accumulation of CCR6^+^ Th17 cells in the bone marrow by inducing CCL20 expression, thereby amplifying IL-17-, TNF-α-, and RANKL-mediated pro-osteoclastogenic signaling. After accumulating in the bone marrow, CCR6^+^ Th17 cells may enhance local inflammation through IL-17 secretion and stimulate RANKL-dependent osteoclastogenesis. Similar gut-derived T-cell trafficking to the bone marrow has been observed in a sustained PTH-induced bone loss model. Following sustained PTH administration, there is a marked increase in the numbers of TNF^+^ T cells and Th17 cells in the small intestine. This change is dependent on the intestinal microbiota, as depletion with broad-spectrum antibiotics suppresses the expansion of TNF^+^ T cells and Th17 cells. Meanwhile, these intestinal T cells exit gut-associated lymphoid tissue and enter the circulation via S1PR1-mediated egress. After entering the bone marrow, TNF^+^ T cells induce CCL20, which subsequently attracts the accumulation of CCR6^+^ Th17 cells (47). The common features in the OVX and continuous PTH models include S1PR1-mediated egress, CXCR3-related entry, and CCL20/CCR6 recruitment. Furthermore, the CCL20/CCR6 axis is not merely a chemotactic signal. Supportive evidence from intestinal inflammation models indicates that CCL20/CCR6 signaling can activate the PI3K-Akt-mTORC1-STAT3 pathway, upregulate RORγt, T-bet, and IL-17A, and promote IL-17 production by CCR6^+^ Th17 cells (61). Thus, the CCL20/CCR6 axis may simultaneously influence both the recruitment of CCR6^+^ Th17 cells and their capacity to produce pro-inflammatory factors such as IL-17.

After CCR6^+^ Th17 cells enter the bone marrow, their pro-osteoclastogenic effect is mainly mediated by the synergistic action of inflammatory factors such as IL-17, TNF-α, and RANKL. Th17 cells can promote RANKL-dependent osteoclastogenesis via IL-17, converting T cell-mediated inflammation into enhanced bone resorption (41). In patients with PMOP, dysbiosis is often accompanied by elevated levels of endotoxin, IL-17A, TNF-α, and IL-6, as well as decreased Treg cells and IL-10 (13). This immune state echoes the bone marrow pro-osteoclastogenic input seen in OVX models, but it does not directly demonstrate that the same gut–bone marrow T cell migration occurs in PMOP patients. The low-grade inflammatory environment associated with PMOP, along with weakened Treg-mediated immunosuppression, may enhance the inflammatory activity of CCR6^+^ Th17 cells in the bone marrow, resulting in persistent amplification of IL-17- and RANKL-mediated osteoclastogenic signaling, which further promotes bone resorption and increases bone fragility.

Bone marrow and callus represent distinct immune microenvironments, and the function of Th17 cells differs between the two. In a standard femoral fracture model, local γδT cells and microbiota-induced gut-derived Th17 cells participate in the early callus IL-17 response. Blocking Th17 egress from the gut or entry into the callus reduces callus Il17a expression and impairs callus formation and mechanical repair (20). This result indicates that the role of gut-derived Th17 depends on their tissue location and the stage of fracture repair. Upon entering the bone marrow, CCR6^+^ Th17 cells are more likely to couple with TNF-α and RANKL pro-osteoclastogenic networks, favoring pre-fracture bone loss and skeletal fragility. In the early callus of standard fracture models, IL-17 may support inflammatory initiation, progenitor recruitment, and callus formation. In PMOP-associated fractures, low bone mass, chronic low-grade inflammation, and insufficient Treg regulation coexist. Whether gut-derived Th17 cells in the callus retain a reparative role, as observed in standard fracture models, or shift toward persistent pro-osteoclastogenic inflammation remains to be validated in PMOP fracture models. Future studies should assess callus Th17/Treg composition, temporal changes in IL-17A expression, Treg function, and mechanical healing quality.

## 5 Microbial metabolites and the immunometabolic environment of osteoporotic fractures

### 5.1 Regulation of Th17/Treg by SCFAs in osteoporotic fractures

Short-chain fatty acids (SCFAs), mainly acetate, propionate, and butyrate, are produced by gut microbial fermentation of dietary fiber and resistant starch. They are involved in processes such as intestinal immune regulation, inhibition of bone resorption, and maintenance of bone formation (21). Compared with the abundance of individual bacterial genera, SCFA profiling more directly reflects one functional dimension of microbial metabolism, although it is also influenced by diet, absorption, and host metabolic status. In patients with PMOP, fecal SCFA profiles are altered, with reduced propionate levels associated with lower lumbar spine and femoral neck BMD (17). Another study in the PMOP population indicated that potential SCFA-producing genera such as Butyricicoccus and Faecalibacterium were reduced, accompanied by increased endotoxin exposure, elevated IL-17A, TNF-α, and IL-6 levels, and a diminished anti-inflammatory response (13). These findings suggest that postmenopausal bone loss is accompanied not only by compositional dysbiosis, but also by reduced SCFA-related metabolic output and a heightened pro-inflammatory immune status. Current human studies mainly support associations among SCFA alterations, bone loss, and inflammatory status, whereas animal studies suggest that improved bone mass is often accompanied by restoration of SCFA-related metabolic function. In OVX rats, intermittent PTH improved bone mass while increasing butyrate-associated taxa such as Ruminococcaceae and Clostridia and enriching the predicted butanoate metabolism pathway (27). Importantly, mechanistic studies in mice have provided stronger evidence that microbiota-derived butyrate is required for the full trabecular anabolic response to intermittent PTH. Microbiota depletion or germ-free conditions impaired the trabecular response to PTH, whereas restoration of physiological butyrate levels rescued this response. Mechanistically, butyrate supported PTH-induced expansion of bone marrow Tregs and subsequent Wnt10b expression by CD8+ T cells, thereby promoting Wnt-dependent bone formation (62). Whether this mechanism operates similarly in human PMOP or contributes to osteoporotic fracture repair remains to be established.

The effects of SCFAs on Th17 and Treg cells are not entirely the same and also differ between intestinal homeostasis and inflammatory states. *In vivo* studies in mice have shown that administration of SCFAs increases the number of colonic Foxp3+ Treg cells, upregulates Foxp3 and IL-10 expression, and enhances colonic Treg function via Ffar2/GPR43 and HDAC-related mechanisms, thereby maintaining mucosal immune homeostasis (63). Studies related to F. prausnitzii further demonstrate that the key anti-inflammatory component in its supernatant is butyrate. Butyrate can inhibit HDAC1, promote Foxp3 expression, and block the IL-6/STAT3/IL-17 pathway, thereby alleviating the Th17/Treg dysregulation (64). Loss of butyrate-producing bacteria may therefore be associated with reduced Foxp3 and IL-10 expression and enhanced Th17-related inflammation (13, 64). SCFAs do not exert anti-inflammatory effects exclusively under all circumstances. *In vitro* and animal experiments have shown that SCFAs can promote IL-10 production via HDAC inhibition and the mTOR-S6K pathway, and can also promote Th1 and Th17 differentiation in specific cytokine milieus (65). Therefore, under the conditions of low-grade inflammation and elevated endotoxin in PMOP, SCFA deficiency is more likely to manifest as diminished Treg regulatory capacity and persistent Th17-related inflammation, rather than a unidirectional change in a single T cell subset.

At the level of bone metabolism, SCFAs can influence bone mass through the combined action of immune cells and bone cells. Butyrate increases both intestinal and bone marrow Treg cells, which in turn promote Wnt10b expression by CD8+ T cells, enhance osteogenic Wnt signaling, and increase bone formation (66). Propionate and butyrate can also directly affect osteoclast metabolism. Bone metabolism studies have shown that propionate and butyrate can increase bone mass and prevent OVX- and inflammation-induced bone loss, as evidenced by decreased CTX-I levels and a reduced number of osteoclasts, with relatively limited changes in bone formation-related indices. Mechanistically, propionate and butyrate induce metabolic changes in early-stage osteoclast precursors, downregulating key osteoclastogenic molecules such as TRAF6 and NFATc1, thereby inhibiting osteoclast differentiation and bone resorption (67). Therefore, SCFAs can both promote bone formation via Treg-associated osteogenic support and directly attenuate osteoclastogenesis. Further studies related to PMOP have shown that reduced levels of *Lactobacillus acidophilus* and butyrate can be associated with bone loss. In OVX mice, supplementation with *L. acidophilus* or butyrate can improve intestinal barrier integrity, inhibit B cell RANKL production and osteoclast activity, and mitigate OVX-induced bone loss (68). Gut-resident regulatory T cells (GTregs) provide new cellular evidence for the involvement of SCFAs in bone metabolic regulation. In OVX mice, intestinal GTregs and peripheral induced Tregs (pTregs) decrease, whereas thymus-derived Tregs (tTregs) increase. pTregs are positively correlated with BMD, tTregs are negatively correlated with BMD, and these changes are accompanied by reduced SCFA levels. GTregs inhibit osteoclastogenesis and promote osteoblastogenesis, and SCFA-pretreated GTregs possess stronger anti-osteoclast activity (69). A decline in SCFAs levels may weaken the immunomodulatory effects mediated by GTregs and pTregs, promote RANKL-related osteoclast activity, and is associated with the process of bone loss in PMOP.

In addition, mouse osteotomy models have shown that antibiotic treatment leads to a decrease in SCFAs and alterations in the microbiota, accompanied by increased circulating TNF-α, IL-17A, and IL-17F, reduced mineral deposition, and a tendency toward delayed bone healing. Butyrate can inhibit osteoclast activity *in vitro* and reduce certain systemic inflammatory markers (70). Recent studies on femoral fractures further demonstrate that antibiotic-induced microbiota depletion can prolong deficits in weight-bearing and functional recovery after fracture, decrease callus bone volume on day 15 post-fracture, and reduce callus BV/TV, Tb.Th, TMD, and BMD on day 28 (71). These findings indicate that the state of the microbiota affects post-fracture inflammatory response, functional recovery, and the mineralization and maturation of the callus. Current research suggests that a decrease in SCFAs may be associated with enhanced post-fracture inflammatory responses, impaired callus formation, and delayed healing, although evidence for SCFA regulation of osteoporotic fracture healing via the Th17/Treg axis remains insufficient. Current evidence more consistently links SCFA deficiency with pro-osteoclastogenic inflammation, weakened Treg-mediated immunoregulation, and reduced osteogenic support.

### 5.2 Regulation of Th17/Treg by tryptophan metabolites in osteoporotic fractures

Tryptophan metabolites participate in immune regulation through the kynurenine pathway, serotonin pathway, and microbiota-derived indole pathway (72). 3-hydroxyanthranilic acid (3-HAA) is a kynurenine-pathway metabolite, whereas indole-3-acetic acid (IAA) and indole-3-propionic acid (IPA) are representative microbiota-associated indole derivatives.These metabolites may act on CD4+ T cells, intestinal epithelial cells, and bone remodeling-related cells through aryl hydrocarbon receptor (AhR)-dependent or immunometabolic mechanisms (72–74). In OVX rats, combined treatment with *Butyricicoccus pullicaecorum* and 3-HAA can improve femoral bone mass and trabecular structure, increase osteoblasts, and decrease osteoclasts, while also reducing the Th17 proportion and increasing the Treg proportion (22). The improvement in bone mass after combined intervention occurred together with partial restoration of Th17/Treg balance, suggesting that tryptophan-related metabolism and microbiota regulation may jointly participate in PMOP-associated osteoimmune dysfunction (22). IPA links tryptophan metabolism to the energy metabolism of CD4+ T cells. Supportive evidence from salt-sensitive hypertension models shows that reduced serum and fecal IPA is accompanied by increased Th17 cells and decreased Tregs, whereas IPA supplementation reduces Th17 responses and promotes Treg differentiation *in vitro* (75). Further mechanistic studies reveal that in CD4+ T cells, IPA can enhance mitochondrial respiration, fatty acid oxidation, and amino acid oxidation via PPARβ/δ while inhibiting glycolysis, thereby reducing the differentiation tendency toward Th1 and Th17 and preserving Treg activity (76). Th17 cells rely primarily on glycolysis for proliferation and effector functions, whereas Treg cells depend more on mitochondrial oxidative metabolism and fatty acid oxidation to maintain immunosuppressive activity (76). IPA-induced metabolic shifts are conducive to inhibiting inflammatory CD4+ T cell responses and promoting the maintenance of Treg-associated immune homeostasis (75, 76).

Microbial indole metabolites can also directly regulate T cell differentiation and activation status via AhR. In inflammatory disease models, indole derivatives such as indoleacrylic acid (IA) and indolealdehyde (IAld) have been reported to activate AhR-related signaling, reduce Th17 differentiation, promote Treg differentiation, and decrease IL-17A levels (77). Indole-3-lactic acid (ILA) and IAA promote Treg expansion through the classical AhR–aryl hydrocarbon receptor nuclear translocator (ARNT)–xenobiotic response element (XRE) signaling pathway, whereas ILA also enhances ZO-1 and occludin expression in intestinal epithelial cells. AhR antagonism attenuates these immunoregulatory and barrier-protective effects (78). Indole-3-carboxaldehyde (ICA) induces *Pik3ip1* transcription through intracellular AhR in T cells and inhibits the PI3K/Akt/mTOR pathway, thereby limiting excessive effector T-cell activation (74). These findings suggest that indole metabolites may act both as intestinal epithelial AhR ligands that support barrier protection and as T-cell AhR ligands that regulate Treg expansion, Th17 restriction, and effector T-cell activation thresholds. In osteoporosis models, IAA and IPA primarily improve intestinal barrier function and gut-bone axis homeostasis through intestinal epithelial AhR signaling (73). In postmenopausal populations and OVX mice, decreased levels of IAA and IPA are associated with bone loss, while supplementation with IAA or IPA can improve intestinal barrier integrity, reduce LPS translocation, and alleviate OVX-induced bone loss (73). Upon deletion of intestinal epithelial AhR, this bone-protective effect is weakened, indicating that the influence of IAA and IPA on the gut-bone axis depends on intestinal epithelial AhR signaling (73). In this model, AhR activation is linked to Wnt/β-catenin-associated barrier repair, increased IL-10, enhanced osteogenesis, and inhibition of osteoclastogenesis. Increased IL-10 and decreased LPS translocation may lower IL-6, TNF-α, and IL-17-associated inflammatory levels, thereby suppressing Th17-related inflammatory responses and supporting Treg function maintenance (73). Functional bacteria or engineered probiotic strategies that enhance tryptophan-derived indole production similarly improve intestinal barrier integrity, reduce systemic inflammation, and mitigate bone loss in preclinical models (79). These findings link abnormal intestinal tryptophan metabolism with barrier impairment, chronic inflammatory activation, and bone loss.

In PMOP patients, IPA levels are reduced, and supplementation of IPA in OVX mice leads to improved bone mass, accompanied by changes in AhR-related Wnt and NF-κB pathways (79). Different tryptophan metabolites exert distinct mechanisms in bone immune regulation. IPA primarily influences CD4+ T-cell function through peroxisome proliferator-activated receptor β/δ (PPARβ/δ)-mediated immunometabolic remodeling, whereas several indole derivatives, including IAA, ILA, IA, IAld, and ICA, act mainly through AhR-related pathways to promote Treg expansion, limit Th17 differentiation, enhance barrier function, or restrain excessive effector T-cell activation (74, 76, 78). Collectively, these effects modulate the Th17/Treg balance and the associated inflammatory milieu, thereby participating in the regulation of bone remodeling. The role of AhR in bone immunity is co-regulated by ligand type, target cells, and the inflammatory microenvironment, and can elicit different immunological and bone metabolic effects under varying conditions (80). For osteoporotic fractures, tryptophan metabolites may affect the pre-fracture bone immune environment and bone remodeling status by regulating intestinal barrier integrity, reducing endotoxin levels, and maintaining Th17/Treg balance. Because Th17 cells and Tregs participate in inflammatory regulation and tissue regeneration during fracture repair, tryptophan metabolite-mediated immunometabolic regulation may influence callus formation, mineralization, maturation, and bone reconstruction, but this remains largely inferential. However, current studies are mainly focused on osteoporosis and gut-bone axis regulation, and direct evidence regarding the role and mechanisms of tryptophan metabolites in PMOP fracture repair remains limited.

### 5.3 Regulation of Th17/Treg by bile acid metabolites in osteoporotic fractures

Bile acid metabolites can influence immune homeostasis by modulating Th17/Treg differentiation. Primary bile acids are converted by the gut microbiota into secondary bile acids with immunomodulatory activity. Among these metabolites, lithocholic acid (LCA) and its derivatives directly regulate CD4+ T-cell differentiation. 3-oxoLCA can bind to the ligand-binding domain of RORγt and inhibit its transcriptional activity, thereby reducing Th17 differentiation and IL-17A expression (81). In contrast, isoalloLCA primarily enhances the Foxp3-dependent program, promoting Treg differentiation through mitochondrial ROS and Foxp3 CNS3 (81). These findings indicate that bile acid metabolites are not only involved in lipid absorption and enterohepatic circulation, but also participate in transcriptional regulation of Th17 and Treg differentiation, thereby shaping inflammatory homeostasis. Multiple human gut Actinobacteria and Firmicutes strains can convert LCA into 3-oxoLCA, and supernatants of Eggerthella lenta cultured with LCA can inhibit naïve CD4+ T cell differentiation into Th17 cells (82). 3-oxoLCA, isoLCA, and their associated biosynthetic genes are reduced in inflammatory bowel disease and are negatively correlated with Th17-associated gene expression (82). These findings suggest that gut microbial changes may reduce LCA-derived secondary bile acids such as 3-oxoLCA and isoLCA, thereby weakening inhibition of Th17 differentiation and favoring a pro-inflammatory T-cell phenotype. In bone-related inflammatory contexts, expanded Th17 cells may increase IL-17 production, promote RANKL-related osteoclastogenesis, and contribute to a bone-resorptive immune environment. Bile acid metabolites play a critical regulatory role in Treg differentiation and maintenance of function. IsoalloLCA can enhance Foxp3 transcription via NR4A1, making the Foxp3 regulatory region more accessible to transcription factors, thus improving Treg differentiation and functional stability (83). In contrast, isoDCA relies more on antigen-presenting cell involvement. It can reduce the immunostimulatory capacity of dendritic cells and promote the generation of Foxp3+ Treg cells in coculture systems (84). Colonization of germ-free mice with engineered isoDCA-producing microbiota leads to an increase in colonic RORγt+ peripheral Treg cells, which is dependent on Foxp3 CNS1-associated genes (84). Bile acids can promote Treg generation and functional maintenance via different pathways. IsoalloLCA primarily enhances Foxp3 transcription and its associated regulatory program, while isoDCA facilitates peripheral Treg expansion by modulating dendritic cell function and the mucosal antigen presentation environment. If bile acid-dependent Treg-supporting mechanisms are weakened under PMOP-related dysbiosis, persistent inflammation and pro-osteoclastogenic cytokine production may be further amplified.

Estrogen deficiency may interact with gut microbiota-bile acid metabolism and immune regulatory networks, thereby contributing to intestinal Th17 inflammation and skeletal fragility. In OVX mice, alginate oligosaccharide (AOS) intervention alters bile acid composition, decreases intestinal Th17 cells and peripheral inflammatory cytokines, and improves intestinal barrier integrity, bone mass, and muscle function. IsoLCA, a bile acid associated with inhibition of Th17 differentiation, is increased after AOS intervention (85). In the context of estrogen deficiency, microbiota-mediated remodeling of bile acid metabolism is closely associated with decreased intestinal Th17 inflammation and reduced systemic inflammatory burden. For osteoporotic fractures, such changes may lower pro-osteoclastogenic immune pressure before fracture and improve the systemic inflammatory state at the time of injury, although this remains to be tested directly. In relation to osteoporotic fractures, the most clearly supported role of bile acid metabolites is the regulation of the pre-fracture immunometabolic state. Insufficiency of 3-oxoLCA and isoLCA may weaken the inhibitory effect on Th17 cells, and insufficiency of isoalloLCA and isoDCA may reduce Treg generation and affect the stability of Foxp3 expression, contributing to persistent low-grade inflammation and pro-osteoclastogenic signaling (81–84). AOS studies in estrogen deficiency models further suggest that improvement of microbial bile acid metabolism can be accompanied by decreases in intestinal Th17, alleviation of systemic inflammation, and improvements in bone loss and bone strength decline (85). However, current studies have not yet simultaneously analyzed changes in bile acid composition, Th17/Treg cell ratios in callus, dynamic changes in IL-17, Treg-mediated tissue repair, and post-fracture mechanical properties in PMOP fracture models. Whether bile acid metabolites can improve post-fracture inflammation resolution, callus mineralization, and remodeling quality still requires direct model validation.

### 5.4 Integrated regulation of the immune microenvironment in osteoporotic fractures by microbial metabolites

In addition to SCFAs, tryptophan metabolites, and bile acid metabolites, TMAO and polyamines represent two contrasting immunometabolic directions through which microbiota-associated metabolites may influence the bone immune environment. TMAO appears to act mainly as a metabolic signal associated with inflammatory amplification. Studies in the elderly have shown that the overall association between TMAO and hip fracture or BMD is not consistent, but in populations with higher BMI, elevated TMAO is associated with increased risk of hip fracture (86). Among overweight and obese individuals, TMAO, the systemic immune-inflammation index (SII), and osteoporosis are also correlated (87). Under metabolically unfavorable conditions, elevated TMAO may be accompanied by chronic low-grade inflammation and immune activation, suggesting its potential involvement in bone metabolic dysregulation. With respect to osteoporotic fractures, TMAO is more likely to exacerbate bone resorption by enhancing pro-inflammatory responses and osteoclastogenic signaling, thereby promoting increased bone fragility and a higher susceptibility to fracture. The adverse effects of TMAO on bone remodeling are mainly reflected in enhanced osteoclastogenesis and impaired osteoblast function. Studies on osteoclasts have demonstrated that TMAO increases ROS production, activates NF-κB signaling, and upregulates osteoclastogenesis-related molecules such as c-Fos and NFATc1, thus promoting osteoclast differentiation and bone resorption (88). Another line of research has shown that TMAO can also impair osteoblast mineralization and mitochondrial energy metabolism via PERK-, ATF4-, and ATF5-related stress responses (89). On the immune level, TMAO can enhance Th17/Th1 polarization, M1 macrophage responses, and NLRP3 inflammasome activation (90). Thus, TMAO may act on both bone cells and immune cells, maintaining a low-grade inflammatory environment through Th17-related immune activation and osteoclastogenic signaling, thereby aggravating dysregulationd bone remodeling. Polyamines, especially spermidine, are closely related to immune homeostasis and play an important role in Treg function regulation. Spermidine can promote the differentiation of Foxp3+ Treg cells and reduce IL-17 production, effects associated with enhanced *Atg5*-dependent autophagy and downregulation of mTOR signaling (91). This mechanism aligns with the feature that Tregs rely on autophagy for function maintenance, whereas Th17 cells depend on inflammatory metabolic programs for expansion. Polyamines can also exert downstream effects on bone cells. Exogenous polyamines promote osteogenic differentiation, enhance mineralization, and reduce osteoclast-related markers such as RANK, NFATc1, and TRAP (92). In the bone environment of aging and oxidative stress, spermidine can also alleviate bone loss via SIRT1/SOD2-mediated antioxidant pathways (93). These results suggest that polyamines may improve the immune environment through Treg support and IL-17 limitation, and may also mitigate dysregulationd bone remodeling by enhancing osteogenesis, suppressing osteoclastogenesis, and exerting antioxidant effects.

Endogenous polyamine levels are jointly influenced by dietary intake, host synthesis, and gut microbial metabolism. Gut microorganisms can synthesize putrescine, spermidine, and spermine, and bacterial polyamines can be absorbed and metabolized by host epithelial cells, thereby contributing to epithelial renewal, macrophage differentiation, and intestinal mucosal homeostasis. Therefore, polyamines should be regarded as part of the microbiota-related immunometabolic network rather than as exclusively host-derived metabolites (94). In osteoporotic fractures, TMAO and polyamines provide two opposing immunometabolic signals: TMAO may amplify metabolic inflammation, Th17-related responses, and osteoclastogenic pressure, while polyamines may support Treg differentiation, constrain IL-17, and enhance inflammatory resolution. Currently, these two molecules are more applicable to explaining low-grade inflammation, Th17/Treg dysregulation, and a pro-osteoclastogenic immune milieu prior to fracture occurrence. Whether they alter the bone callus Th17/Treg composition, inflammation resolution, mineralization and remodeling, or mechanical healing quality after fracture remains to be directly demonstrated in PMOP fracture models.

## 6 Translational strategies targeting the gut microbiota-Th17/Treg-callus axis in osteoporotic fracture repair

### 6.1 Role of gut microbiota intervention in the management of osteoporotic fracture risk

Management of osteoporotic fractures should not begin only after fracture occurrence or focus solely on local repair. In osteoporosis, the combination of low bone mass, deterioration of bone microarchitecture, decreased bone strength, and chronic inflammation collectively increases fracture risk and also affects post-fracture resolution of inflammation, callus formation, and callus remodeling. Experimental studies suggest that osteoporosis can be associated with insufficient callus formation, reduced callus mineral density, decreased mechanical strength, and delayed cellular differentiation. Clinically, osteoporotic patients may face challenges such as fixation difficulty, delayed healing, and increased risk of subsequent fracture (95). Therefore, the value of gut microbiota intervention in osteoporotic fracture management may lie mainly in reducing pre-fracture skeletal fragility and improving the immunometabolic microenvironment during the peri-fracture period. Probiotics, prebiotics, and natural bioactive compounds can help improve pre-fracture bone mass, bone microarchitecture, and bone strength by repairing the intestinal barrier, reducing LPS-related inflammation, restoring metabolites such as SCFAs and bile acids, alleviating Th17/Treg dysregulation, and inhibiting RANKL-related osteoclastic activity (96).

#### 6.1.1 Functional strains and probiotic intervention

A narrative review has proposed that probiotics may have potential value in fracture care by modulating inflammation, pain, and bone regeneration, but direct clinical evidence remains limited (97). The key to precise probiotic interventions lies in selecting functional strains that can stably modulate barrier function, LPS exposure, microbiota metabolites, and Th17/Treg dysregulation. Guo et al. found in OVX rats that Lactobacillus rhamnosus GG can improve bone microarchitecture, bone mechanical properties, and bone turnover markers, as well as repair intestinal barrier damage and regulate both intestinal microbiota composition and intestinal and bone marrow Th17/Treg dysregulation. After LGG intervention, TNF-α and IL-17 levels decreased in the colon and bone marrow of OVX rats, while IL-10 and TGF-β increased, indicating that this strain can target both intestinal mucosal inflammation and the bone marrow pro-osteoclastogenic immune environment (16). Mechanistic studies of butyrate further show that this microbial metabolite expands intestinal and bone marrow Tregs and promotes CD8+ T-cell Wnt10b expression, thereby activating osteogenic Wnt signaling (66). Together, the LGG and butyrate studies suggest that the bone-protective effects of microbiota-based interventions may arise not merely from changes in microbial abundance, but from interactions among microbial metabolites, Treg regulation, and osteogenic signaling. In osteoporotic fractures, the significance of such strains lies in reducing the pro-osteoclastogenic inflammatory burden prior to fracture and providing a more stable systemic immune environment for callus formation after fracture, though whether they can directly improve callus mineralization, callus remodeling, and the mechanical quality of healing still requires validation in osteoporotic fracture models. In recent years, probiotic interventions have advanced to the level of strain selection and immune function modulation. Studies on *Bacillus coagulans* have shown that, under PMOP conditions, endotoxin-producing bacteria increase, SCFA-producing bacteria decrease, accompanied by dysregulation of the Breg-Treg-Th17 axis and inflammatory bone loss. Supplementation with *Bacillus coagulans* can improve BMD, bone strength, and bone microarchitecture, and enhance the immunoregulatory and anti-osteoclastogenic potential of Breg (60). *Bifidobacterium longum* has also been shown to increase BMD, bone strength, and bone microarchitecture in OVX mice, and to impact the Breg-Treg-Th17 axis by enhancing the anti-osteoclastogenic and immunoregulatory potential of Bregs, reducing TNF-α, IL-6, and IL-17, and increasing IL-10 and IFN-γ (98). Research on *Parabacteroides distasonis* showed that this strain can improve trabecular bone microarchitecture and bone strength in the distal femur of OVX rats, decrease serum LPS, increase expression of ZO-1 and occludin, and shift Th17/Treg-related indices in mesenteric lymph nodes and bone marrow regions toward a low-inflammatory state (54). Further studies on *Lactobacillus acidophilus* suggest that in the PMOP state, homeostasis of Treg subsets in the gut and bone marrow is impaired, and *Lactobacillus acidophilus* can restore the balance between gut-resident RORγT−pTreg and Th17 cells and enhance the anti-osteoclastogenic potential of RORγT−pTreg, with a mechanism involving butyrate (99). Therefore, the goal of probiotic intervention should not only be to increase BMD, but also to reduce the pro-inflammatory, pro-osteoclastogenic state preceding fracture, maintain barrier integrity and T cell immune homeostasis, and reduce the systemic inflammation that may be encountered during post-fracture repair.

#### 6.1.2 Prebiotics and natural product interventions

Prebiotics and natural product interventions do not directly supplement a single functional strain, but instead indirectly alleviate LPS-related inflammation and Th17/Treg dysregulation by reshaping gut microbiota composition, altering levels of microbiota-associated metabolites, and repairing the intestinal barrier. Compared with single probiotics, such strategies are more suitable for long-term modulation of the gut-bone axis environment in osteoporosis, and may participate in osteoporosis fracture risk management by reducing pro-osteoclastogenic inflammation and improving bone mass and bone strength. Ai et al. found in OVX rats that 12-week intervention with 8% KOS alleviated bone loss and improved trabecular structure and femoral biomechanical properties. KOS promoted the growth of *Bifidobacterium longum*, repaired the intestinal barrier, reduced pro-inflammatory factor levels, and restored the Treg/Th17 balance, thereby decreasing inflammation-related osteoclastic activity (53). This result suggests that, therefore, KOS is better understood as a prebiotic-like intervention that synergistically attenuates inflammatory bone resorption through microbiota expansion, barrier repair, and restoration of Treg/Th17 homeostasis, rather than exerting bone-protective effects solely by promoting calcium absorption or increasing SCFAs. For osteoporotic fractures, such long-term interventions are more suitable for pre-fracture risk control and prevention of refracture, especially in elderly or postmenopausal populations with concomitant gut dysbiosis, low-grade inflammation, and inadequate nutrient intake. Different prebiotics may modulate osteoimmunity through different metabolic modules. Studies by Zhang et al. have shown that alginate oligosaccharides improve bone mass, muscle function, and intestinal barrier integrity in OVX mice, and reduce the proportion of intestinal Th17 cells and levels of peripheral inflammatory factors. AOS intervention is accompanied by bile acid remodeling and increased isoLCA, a bile acid associated with inhibition of Th17 differentiation, suggesting that prebiotics may modulate Th17 inflammation through microbiota-mediated bile acid transformation (85). Given that sarcopenia, fall risk, and systemic inflammation collectively influence the incidence of fractures and postoperative recovery, prebiotic interventions may simultaneously improve bone mass, muscle function, and immunoinflammatory status. Natural products and compound preparations can also exert long-term regulatory effects via the gut-bone axis. Hao et al. reported that Qing’e Pills improved BMD and bone biomechanical properties in OVX rats, decreased serum LPS, restored gut barrier integrity, downregulated RORγt, IL-17A, and the Th17 proportion, while upregulating Foxp3, IL-10, TGF-β, and the Treg proportion (100). This study also demonstrated that Qing’e Pills increased the abundance of Lactobacillus and influenced Th17/Treg dysregulation in the gut-bone axis via microbiota-associated endogenous metabolites (100). Wang et al. found in OVX mice that ginkgolide B alleviated estrogen deficiency-induced bone loss, enhanced gut barrier integrity, repro-inflammatorymatory markers, and restored Th17/Treg balance in mesenteric lymph nodes and spleen, while increasing beneficial bacteria such as Lactobacillaceae and Prevotellaceae (101). Clinical studies suggest that probiotic supplementation may help increase lumbar spine and hip BMD in postmenopausal women and reduce CTX and BALP levels, but its efficacy is more evident in individuals with low bone mass than in osteoporotic patients, and current studies have not used fracture healing quality as the primary endpoint (102). Synbiotics, combining functional strains with utilizable substrates, are entering clinical research design for bone health and metabolic health in postmenopausal women, providing a translational direction for long-term management in high-risk populations before fracture. However, their effects on osteoporotic fracture risk, peri-fracture immune status, and fracture healing quality still require further validation (103). Collectively, these lines of evidence indicate that interventions with prebiotics or natural products such as KOS, AOS, Qing’e Pills, and ginkgolide B may alleviate osteoclastogenic responses and declines in bone strength under estrogen deficiency by remodeling microbiota composition, repairing gut barrier function, reducing LPS-related inflammation, and modulating Th17/Treg homeostasis or Th17-associated inflammation. Therefore, such interventions are more suitable as long-term risk management strategies for osteoporotic fractures, aiming to reduce inflammatory burden and underlying bone fragility prior to fracture. Whether they can further improve callus formation, mineralization and remodeling, and mechanical healing quality after fracture requires validation in osteoporotic fracture models and peri-fracture clinical studies.

### 6.2 From pre-fracture immune regulation to the endpoint of osteoporotic fracture healing

The therapeutic goals for osteoporotic fractures are not limited to preserving bone mass and reducing fracture risk, but also emphasize effective post-fracture repair and functional recovery. Improvements in bone mass and systemic inflammation do not necessarily indicate successful fracture repair. Callus quality, dynamic immune-cell changes, and tissue regeneration during healing are also decisive for the final outcome. The translational value of fracture-related interventions should be reflected in increased callus volume and mineralization, coordinated transition from cartilaginous to bony callus, improved immune-cell composition and function within the callus, accelerated remodeling, and enhanced mechanical healing quality. After fracture, callus T-cell composition, temporal IL-17 changes, Treg reparative function, cartilage-to-bone transition, callus mineralization and remodeling, and mechanical healing quality become key endpoints for evaluating therapeutic relevance (95, 104). Animal models of fracture indicate that perifracture immunomodulation can directly affect callus formation and the healing process. A2A receptor activation increases callus volume and mineralized tissue volume, reduces IL-6 levels in blood and fracture tissue, increases Tregs, and decreases Th17 cells. Exogenous IL-6 weakens these healing-promoting effects (104). The Th17/Treg status during the peri-fracture period does not merely reflect inflammatory intensity, but can be therapeutically modulated to create an immune microenvironment conducive to repair. Fracture models show that microbiota-induced gut Th17 cells can egress from the intestine and home to the callus, and that blocking either their egress or recruitment to the callus impairs fracture repair (20). During fracture healing, Tregs not only control inflammation but also directly influence regeneration. Studies have shown that CCR8+ Treg cells can promote skeletal stem cell accumulation and osteogenic differentiation through secretion of PGRN, thus supporting fracture repair (19). Research on IL-17RA in Prx1+ mesenchymal cells demonstrates that IL-17 signaling is involved in the regulation of the cartilage-to-bone transition and the function of periosteal-derived cells, with its effects dependent on the repair stage and cellular targets (40). These studies demonstrate that the roles of Th17 and Treg cells in fracture repair differ across distinct healing phases and cell types, and their influence on callus formation, endochondral ossification, and bone remodeling collectively determines the ultimate healing quality.

Immunoregulatory materials offer local intervention strategies for this translational process. After implantation, bone repair materials interact with macrophages, T cells, and the local immune microenvironment. Persistent inflammation can delay healing, whereas appropriate immunomodulation supports bone regeneration (105). Within this framework, Th17/Treg regulation has become an important immunological target in bone tissue engineering, with nanostructures, hydrogels, and exosomes being used to limit Th17-associated inflammation or promote Treg-mediated repair (106). Demineralized dentin matrix (DDM) promotes Treg differentiation through IDO1, enhances BMSC osteogenic activity, and increases BMD, BV/TV, and osteogenesis-related markers in a rabbit mandibular defect model. IDO1 inhibition attenuates these reparative effects (107). Mn-doped silicate hydroxyapatite nanowires further show that local materials can target adaptive T-cell immunity. This material increases CD4+ T-cell recruitment, elevates Th2-related IL-4 and Gata3, and reduces Th1-related IFN-γ and T-bet. CD4+ T-cell depletion significantly weakens bone defect repair (108). These results indicate that local materials are already capable of shaping the bone repair microenvironment through T cell polarization, though the primary evidence still derives from bone defect and bone tissue engineering contexts. For osteoporotic fractures, a more critical issue is whether these local T cell regulatory strategies can, together with upstream microbiota interventions, jointly improve callus immunity, mineralization remodeling, and mechanical healing. Oral gut-targeted materials provide support for this translational process by modulating the systemic immunometabolic milieu. In OVX models, colon-targeted postbiotic nanoparticles deliver butyrate, restore ZO-1 and claudin-1 expression, reduce LPS, TNF-α, and IL-6, and improve bone formation, bone resorption status, bone mass, and femoral mechanical properties (109). Nano-armored probiotics improve the gastrointestinal survival and colonization efficiency of Lactobacillus rhamnosus, alleviate intestinal inflammation, oxidative stress, and barrier damage, and enhance indices related to microbiota, SCFAs, and bone remodeling (110). Compared to local bone repair materials, oral delivery is more suited for long-term modulation of the microbiota, barrier, LPS, and systemic inflammation, while local materials are better suited for regulating the immune and osteogenic microenvironment at the fracture site. Systemic microbiota modulation and local immunoregulation may act at different levels of fracture repair. The former improves the immune and inflammatory background before and around fracture, whereas the latter directly shapes the cellular microenvironment during callus formation and remodeling. Their ultimate value is reflected in improvements in the immune status of the callus, degree of mineralization maturation, and mechanical healing quality. Future osteoporotic fracture models should integrate systemic and local endpoints, including microbiota composition, intestinal barrier integrity, LPS exposure, serum metabolites, bone marrow and callus Th17/Treg ratios, temporal IL-17 dynamics, Treg-associated reparative molecules such as PGRN, micro-CT-based callus architecture, histological mineralization, and callus mechanical strength. These endpoints would allow a more direct evaluation of how microbiota modulation and T-cell immune changes influence fracture healing quality. A proposed translational framework for microbiota-centered immunomodulation in osteoporotic fracture repair is shown in **Figure 3**.

**Figure 3:**
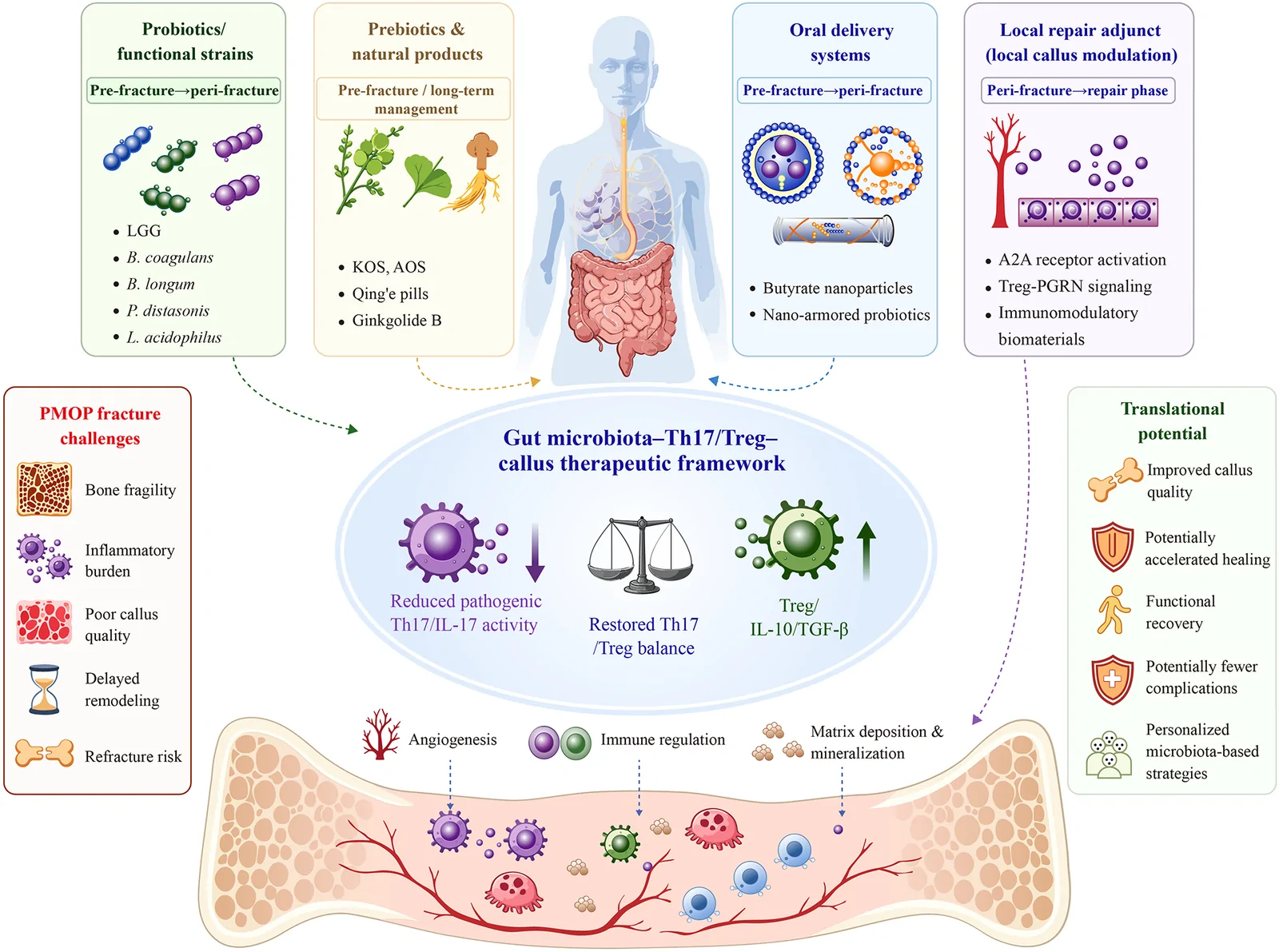
Translational strategies targeting the gut microbiota–Th17/Treg–callus axis in osteoporotic fracture repair. PMOP-associated fractures are characterized by skeletal fragility, inflammatory burden, poor callus quality, delayed remodeling, and increased refracture risk. Gut microbiota-based interventions may act at different stages of fracture management. Probiotics and functional strains are positioned mainly from the pre-fracture to peri-fracture period, whereas prebiotics and natural products are more suited to long-term pre-fracture risk management. Oral delivery systems may also span the pre-fracture to peri-fracture period by improving the systemic immunometabolic environment through intestinal barrier repair, reduced LPS-related inflammation, restoration of microbial metabolites, decreased pathogenic Th17/IL-17 activity, and enhanced Treg-associated IL-10 and TGF-β signaling. Local repair adjuncts, including A2A receptor activation, Treg-PGRN signaling, and immunomodulatory biomaterials, are positioned primarily during the peri-fracture and subsequent repair phases, where they may regulate the callus immune microenvironment during angiogenesis, matrix deposition, mineralization, and remodeling. Together, systemic microbiota modulation and local callus immunoregulation represent a potential translational strategy for improving callus strength, healing efficiency, functional recovery, and personalized microbiota-based management. These intervention windows are conceptual and reflect the predominant roles suggested by current evidence rather than established clinical treatment schedules, and direct validation in PMOP fracture models and peri-fracture clinical studies remains necessary.

## 7 Discussion and perspectives

Osteoporotic fracture should not be viewed solely as a mechanical consequence of low bone mass, but as a repair challenge shaped by skeletal fragility, microbiota-related metabolic disturbance, intestinal barrier dysfunction, and osteoimmune dysregulation. Current evidence suggests that PMOP-associated gut dysbiosis is accompanied by increased LPS exposure, enhanced pro-inflammatory cytokine signaling, and Th17/Treg dysregulation, while selected probiotics and microbiota-targeted interventions can improve bone mass, bone microarchitecture, and T-cell immune homeostasis. Prospective cohort and genetic prediction studies further link gut microbial features to osteoporotic fracture risk (13, 16, 28, 29). Fracture repair studies provide an additional layer of evidence. In standard fracture models, gut-derived Th17 cells can enter the callus and participate in repair, IL-17RA signaling can influence the cartilage and bone composition of the callus, and Tregs can support skeletal stem cell recruitment and osteogenic differentiation through PGRN-related reparative functions (19, 20, 40). These findings suggest that the gut microbiota-Th17/Treg axis may connect pre-fracture skeletal fragility, peri-fracture immune status, and post-fracture repair quality. Compared with gut-bone axis studies centered mainly on BMD, bone turnover markers, and bone metabolic pathways, osteoporotic fracture research requires greater attention to callus formation, mineralization, remodeling, and mechanical healing quality.

The major limitation at present is that relevant evidence remains distributed across PMOP bone-loss models, standard fracture models, microbiota intervention studies, and local bone regeneration models. Studies that simultaneously incorporate an osteoporotic background, standardized fracture models, gut microbiota composition, barrier function, microbial metabolites, bone marrow and callus Th17/Treg phenotypes, and mechanical healing endpoints remain scarce (95).Therefore, current evidence does not yet establish that microbiota alterations directly determine osteoporotic fracture healing quality through the Th17/Treg axis. Clinical studies also focus mainly on BMD, bone turnover markers, and microbiota composition, whereas fracture incidence, refracture rate, postoperative healing time, functional recovery, and callus quality have not been sufficiently evaluated (102, 103). Several interventions have shown repair-related benefits in fracture, aged fracture, osteoporotic bone repair, or osteoporotic bone defect models, including Jinkui Shenqi Pill, local adenosine delivery, inhibition of inflammatory osteoclasts, and immunomodulatory hydrogels (111–116). These studies support the therapeutic plasticity of callus formation, callus remodeling, and local regeneration, but they do not establish that these effects are mediated by the gut microbiota or the Th17/Treg axis. Future studies should evaluate microbiota composition, microbial metabolites, intestinal barrier function, LPS exposure, bone marrow and callus T-cell phenotypes, temporal IL-17 dynamics, Treg-associated reparative molecules, callus architecture, mineralization, remodeling, and mechanical outcomes within the same osteoporotic fracture model. This integrated design would help determine whether the gut microbiota-Th17/Treg axis actively participates in osteoporotic fracture repair rather than merely reflecting systemic bone loss or inflammation. IL-17- and Treg-related effects are stage-dependent and context-dependent, and intervention strategies should not be simplified as uniform Th17 inhibition or Treg enhancement. Instead, therapeutic timing and application should be defined according to fracture healing stage, inflammatory intensity, cellular source, and osteoporotic status. Clinical translation will also require attention to the heterogeneity of postmenopausal women, older fracture patients, and individuals at high risk of refracture, together with standardized strain selection, dose, duration, and combined medication strategies, while controlling confounding factors such as diet, antibiotics, anti-osteoporotic drugs, and surgical variables. The gut microbiota-Th17/Treg axis cannot explain the entire pathobiology of osteoporotic fracture, but it provides a mechanistic framework linking pre-fracture fragility, peri-fracture immune status, and post-fracture repair quality. Multi-endpoint evaluation and stratified intervention studies centered on this axis may help move osteoporotic fracture management beyond isolated antiresorptive or anabolic treatment toward an integrated strategy that considers intestinal microecology, immune homeostasis, and callus healing quality.
